# Investigation on the Formation Mechanism of Double-Layer Vertically Aligned Carbon Nanotube Arrays via Single-Step Chemical Vapour Deposition

**DOI:** 10.1007/s40820-016-0113-5

**Published:** 2016-10-07

**Authors:** Shoumo Zhang, Deli Peng, Huanhuan Xie, Quanshui Zheng, Yingying Zhang

**Affiliations:** 1 Center for Nano and Micro Mechanics, School of Aerospace Engineering, Tsinghua University, Beijing, 100084 People’s Republic of China; 2 Department of Chemistry, Tsinghua University, Beijing, 100084 People’s Republic of China; 3 State Key Laboratory of Tribology and Applied Mechanics Laboratory, Tsinghua University, Beijing, 100084 People’s Republic of China

**Keywords:** Synthesis, Vertically aligned carbon nanotube arrays, CVD, Double-layer

## Abstract

**Abstract:**

The mechanism for the formation of double-layer vertically aligned carbon nanotube arrays (VACNTs) through single-step CVD growth is investigated. The evolution of the structures and defect concentration of the VACNTs are tracked by scanning electron microscopy (SEM) and Raman spectroscopy. During the growth, the catalyst particles are stayed constantly on the substrate. The precipitation of the second CNT layer happens at around 30 min as proved by SEM. During the growth of the first layer, catalyst nanoparticles are deactivated with the accumulation of amorphous carbon coatings on their surfaces, which leads to the termination of the growth of the first layer CNTs. Then, the catalyst particles are reactivated by the hydrogen in the gas flow, leading to the precipitation of the second CNT layer. The growth of the second CNT layer lifts the amorphous carbon coatings on catalyst particles and substrates. The release of mechanical energy by CNTs provides big enough energy to lift up amorphous carbon flakes on catalyst particles and substrates which finally stay at the interfaces of the two layers simulated by finite element analysis. This study sheds light on the termination mechanism of CNTs during CVD process.

**Graphical Abstract:**

The mechanism for the formation of double-layer vertically aligned carbon nanotube arrays (VACNTs) through single-step CVD growth was investigated. The growth of the second CNT layer lifts the amorphous carbon coatings on catalyst particles and substrates.
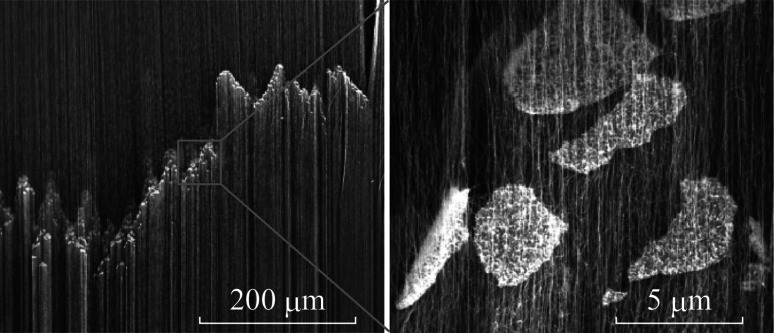

**Electronic supplementary material:**

The online version of this article (doi:10.1007/s40820-016-0113-5) contains supplementary material, which is available to authorized users.

## Introduction

Carbon nanotubes (CNTs) have been widely investigated since the report by Iijima in 1991 [1], due to their unique structures and superior properties [2–6]. The practical application of CNTs depends on the development of synthesis approaches. Most CNT production, which is mainly unorganized CNTs and has limited properties, is currently mainly utilized in bulk composite materials and thin films. In contrast, organized CNT architectures, such as horizontally aligned CNT arrays [7–14] and vertically aligned CNT (VACNT) arrays, have superior properties which promise new functionalities and applications [15, 16]. In a VACNT array, all the CNTs are perpendicular to the surface and aligned very well, forming a forest-like structure. Due to their unique structures, VACNTs have been suggested for applications in energy-absorbing, thermal management, highly specular absorbing, electromagnetic shielding coatings, super strong fibres, novel nano-composites, desalination membranes, and high-performance electrodes [17–28].

The main technique to grow VACNTs is chemical vapour deposition (CVD). CVD is considered as the most advantageous method for the synthesis of VACNTs due to the fact that the growth condition can be easily controlled, and the CVD process can be integrated into the standard lithographic methods, which is suitable for chip fabrication. Typically, the CNTs in a VACNT array, if using a predeposited metal film as the catalysts, are continuous from the bottom to the top. However, the growth of multilayer VACNTs, in which the CNTs are not continuous, has also been reported [18, 29, 30]. Stacked multiple layers of VACNTs formed through multistep CVD processes were reported by Ajayan et al. [30]. Multilayer aligned CNTs were also synthesized by Martine et al. using an aerosol-assisted catalytic CVD process [29].

Multilayer VACNTs have diverse applications such as acting as composite reinforcements, p-n junctions for electronic devices, and allowing the fabrication of complex multilayer nanotube structures [31]. However, for practical applications, single-step CVD methods, which are simple and cost effectively compared to multisteps, are preferred. Zhang et al. reported the growth of double-layered VACNT arrays via single-step CVD method [18]. Although it was proposed that the growth of the double-layered VACNTs by single-step CVD should originate from the deactivation and reactivation of the catalysts, experimental evidence and better understanding on the mechanism are still lacked.

This work systematically investigated the evolution of the structure of VACNTs with the growth duration and proposed the mechanism for the formation of double-layered VACNTs through single-step CVD. There are amorphous carbon flakes between the top and the bottom CNT layers, which should be responsible for the termination of the first CNT layer. Besides, by controlling the growth parameters, the height of the bottom layer in the double-layered VACNTs can reach millimetre scale. These results shed new light on the termination of growth of CNTs and the formation mechanisms of multilayer VACNTs via single-step CVD.

## Methods

VACNTs were grown in a quartz tube furnace via CVD method. Si wafers coated with silicon oxide (~1 μm) were used as substrates. A thin layer of Fe (~2 nm) supported with Al_2_O_3_ (~10 nm) was used as the catalysts. The furnace temperature was ramped to 750 °C in 10 min with flow of Ar (140 sccm) and H_2_ (20 sccm). Then C_2_H_4_ (35 sccm) was introduced as the carbon source for the growth of CNTs. Growth time of 15, 30, 60, and 120 min were applied to obtain different samples. The growth was terminated by turning off C_2_H_4_ gas and cooling down the furnace under the protection of H_2_ and Ar.

## Results and Discussion

**Fig. 1:**
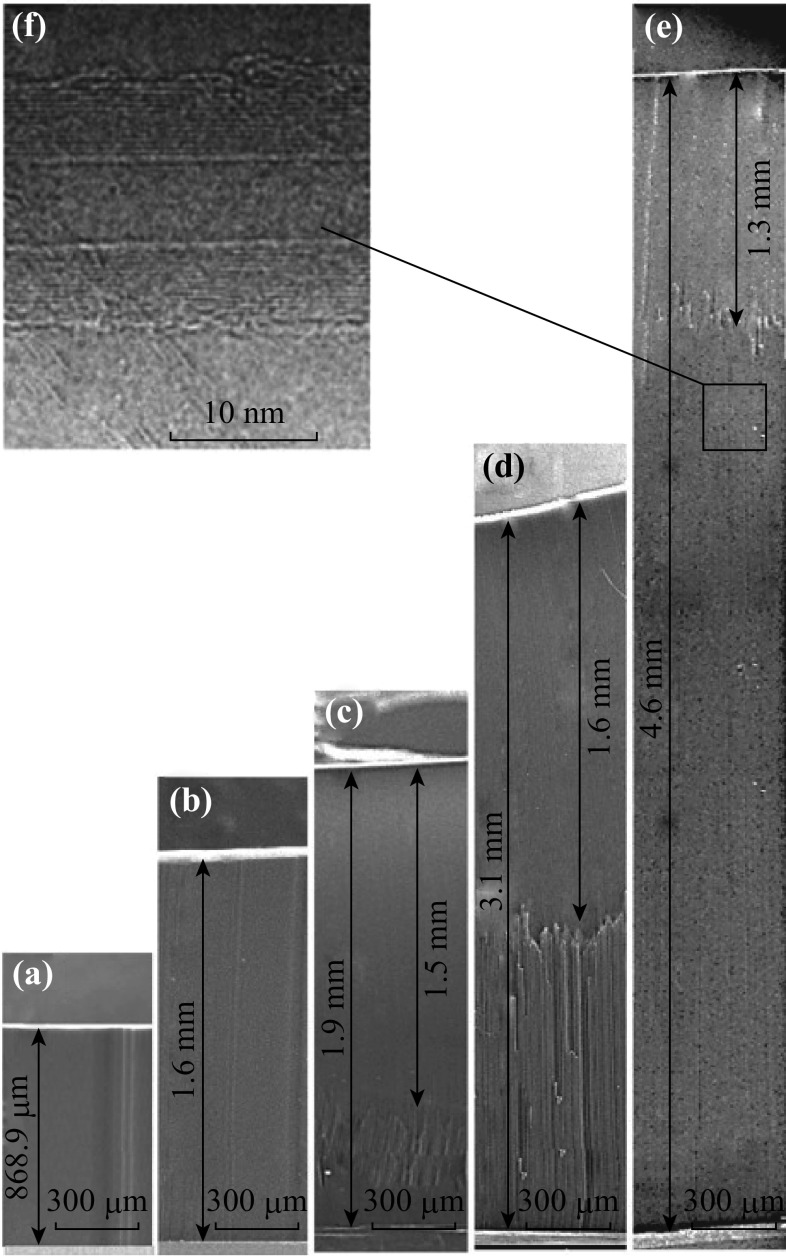
Side-view SEM images of VACNTs obtained by different growth time. **a** 15 min (868.9 μm in height). **b** 30 min (1.6 mm in height). **c** 30 min (1.9 mm in height and the height of the top layer is 1.5 mm) **d** 60 min (3.1 mm in height and the height of the top layer is 1.6 mm) **e** 120 min (4.6 mm in height and the height of the top layer is 1.3 mm) **f** TEM analysis of CNTs

Figure 1 shows typical side-view SEM images of the VACNTs grown with different time. It is clearly observed that different growth time led to VACNTs with different heights and morphologies. The height of VACNTs was increased almost linearly from the height of 868.9 μm for 15 min, the height of 1.6–1.9 mm for 30 min, and then to the height of 3.1 mm for 60 min. However, the linear growth broke down when the growth duration lasted for 120 min, which resulted a height of 4.6 mm. This indicates that the growth rate can remain for a certain time then it decreases with elongated time. As shown in (Fig. 1a), the sidewall of VACNTs grown by 15 min is continuous. However, as shown in Fig. 1b, c, for the growth time of 30 min, there are both of one layer of VACNTs and double-layer VACNTs produced, indicating that 30 min is a critical growth time point for the formation of the second-layer VACNTs during the process. Figure 1d, e shows that the VACNTs grown through 60 and 120 min are both double-layer VACNTs. It is clearly illustrated that the height of the top layer is in the range of 1.3–1.6 mm, and the height of the bottom layer is continuously increasing with the increase of growth time. The morphology of VACNTs grown by CVD method is known to be significantly affected by the growth conditions, such as the precursors, catalysts, and gas pressure [32]. It has been previously reported that double-layered carbon nanotube arrays could be formed through one-step CVD for growth time longer than 3 h. Although the growth time reported here is obviously shorter than the reported 3 h, the height of the bottom layer in our obtained VACNTs is much larger than the previously reported ones [18], indicating a faster growth rate resulted from the optimized process parameters in this work. The CNTs in this study are mainly multiwalled CNTs, as shown in the transmission electron microscope (TEM) image (Fig. 1f).

**Fig. 2:**
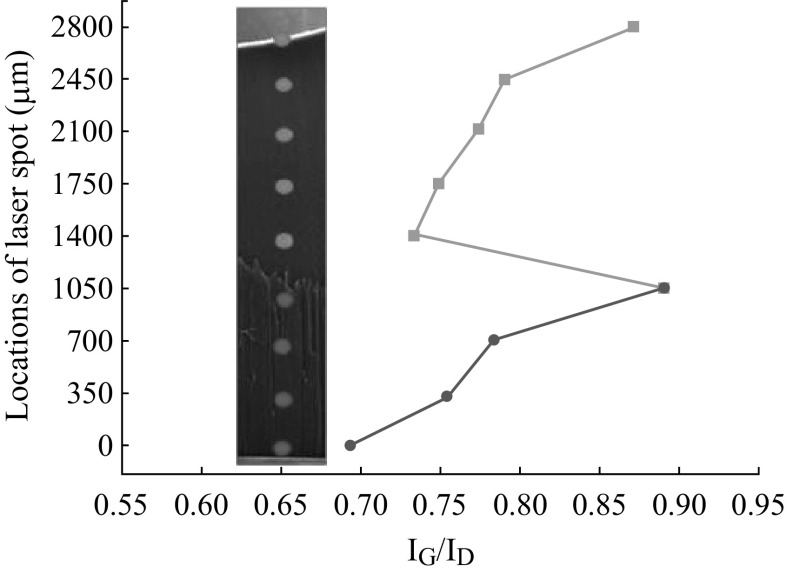
Variation of intensity ratio of G/D-band (*I*_G_/*I*_D_) of Raman spectra along the vertical direction of a double-layer VACNT array

A series of Raman spectra of the double-layer VACNTs were recorded when the laser spot was focused on the sidewall of VACNTs and moving along the axial direction of CNTs. Raman spectra were collected under ambient atmosphere using a 633 nm laser. The step size was 300 μm and the diameter of the laser spot was ~1 μm. It is well known that Raman G-band and D-band are the two main bands for multiwalled CNTs. G-band indicates the level of graphitized carbon and D-band demonstrates disordered amorphous carbon [33]. The intensity ratio of G-band to D-band (*I*_G_/*I*_D_) could be used to study the quality of the CNTs. The variation of *I*_G_/*I*_D_ along the axial direction of the VACNTs was studied. Figure S6 provides the typical Raman spectrum data in the Supplementary Information, and Fig. 2 shows the decreasing of the intensity of *I*_G_/*I*_D_ in both the top and the bottom layer with the growth of CNTs. However, a sharp increase of *I*_G_/*I*_D_ across the interlayer was apparent. The decreasing of the intensity of *I*_G_/*I*_D_ suggests that the concentration of amorphous carbon or defects decreases [18]. The decreasing of *I*_G_/*I*_D_ of both the top layer and the bottom layer corresponds to the deactivation of catalyst particles as the growth time increases. The sharp increase of *I*_G_/*I*_D_ at the interface of the two layers indicates that the bottom layer is a new VACNT layer.

**Fig. 3:**
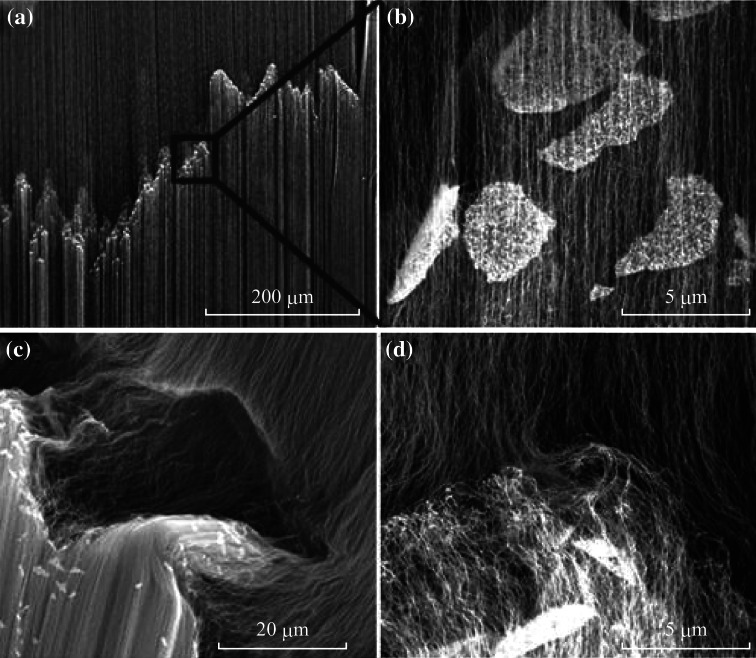
**a** SEM image showing the interface between the top layer and the bottom layer of a double-layer VACNT array. **b** Magnified SEM image of (**a**). **c** SEM image showing the facture interface between top layer and bottom layer for double-layer VACNTs after pulling test with nippers. **d** Magnified SEM image of the interface

The interface between the top layer and the bottom layer of a VACNT array was further characterized by SEM, as shown in Fig. 3. The interfaces were not flat and uniform. In the enlarged SEM image (Fig. 3b), it can be seen that there are some flakes with size of several micrometres at the interface. The top end of the bottom layer VACNTs is connected to the flakes. The bottom end of some top layer CNTs is also connected with the flakes. An attempt was made to separate the top and the bottom CNT layers by pulling the two layers toward opposite directions with nippers. Figure 3c, d demonstrates the fractured interfaces, indicating that the CNTs in the VACNTs are not continuous since the two layers could be separated at the interface. It is also showed that the CNTs at the interface are curlier than the other parts of VACNTs.

**Fig. 4:**
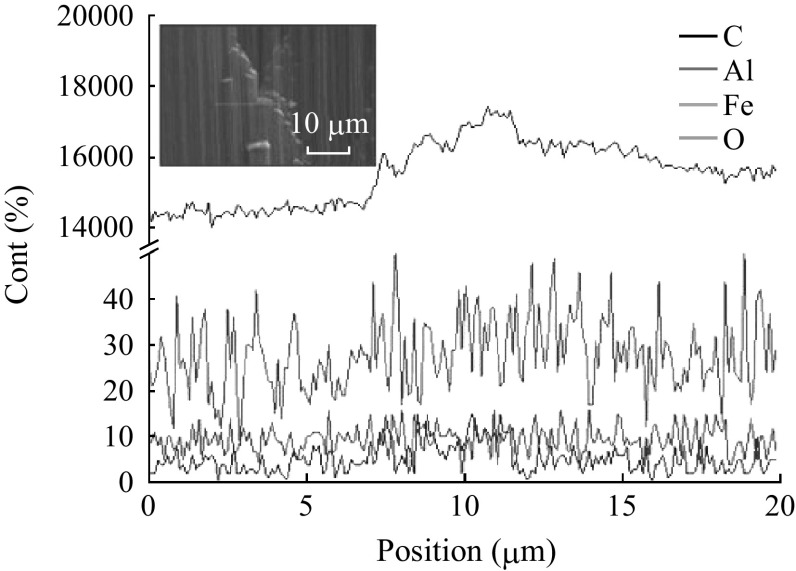
Quantitative analysis of C, O, Al, and Fe elements on the interface between the top layer and the bottom layer as characterized by one-line scan electron microprobe technique

Electron microprobe was used to analyse the chemical element on the interface of the double-layer VACNTs. Elements of C, O, Al, and Fe, which are involved in our CVD process, are qualitatively analysed through one-line scan across the interface. Figure 4 shows that the contents of O, Al, and Fe are all below the detection limit while only the content of C is high, indicating the absence of these elements at the interface. It is obviously that the flakes at the interface are composed of pure carbon without trace of metal, indicating that the metal catalyst layer stays on the substrate during the whole process. This is in consistent with the base-growth mode of VACNTs [18].

**Fig. 5:**
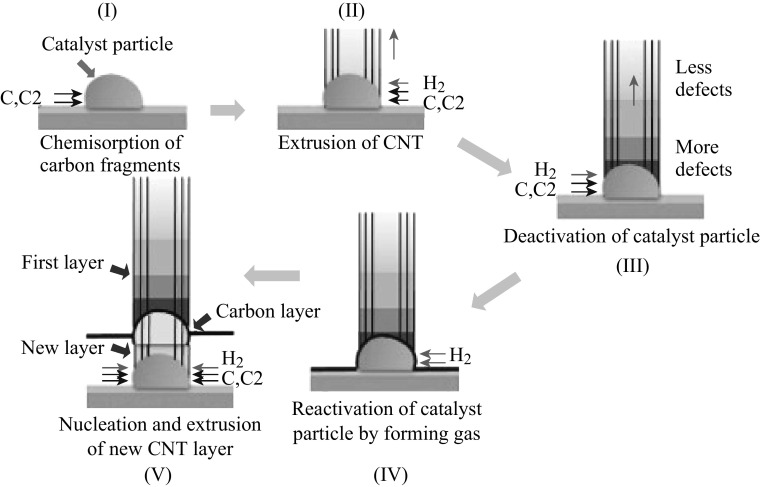
Illustration showing the formation mechanism of double-layer VACNT arrays by single-step CVD method

According to the above observations, we proposed the mechanism for the growth of double-layered VACNTs via single-step CVD method, which is illustrated in Fig. 5. At the beginning of the growth process, carbon source (C_2_H_4_) is pyrolysed into carbon fragments through the high-temperature treatment, which could be absorbed on the surface of the metal catalyst particles (Fe particles). It is well known that the growth of CNTs with metal catalysts in a CVD process usually obeys vapour–liquid–solid (VLS) mechanism [34, 35]. The process could be divided into three stages: absorption of carbon fragments in vapour phase on the catalyst nanoparticle surfaces, diffusion of carbon atoms in the melted catalyst nanoparticles, and extrusion of CNTs from the metal nanoparticles [36, 37]. With the prolonged growth, the quality of active positions on catalyst particles will degrade, even though the feeding rate of carbon precursor is fixed [38–42]. Hence, with the growth time increasing, both the top layer and the bottom layer demonstrate increased defects and degraded qualities since increased amorphous carbon will accumulate on the surface of catalyst surfaces. The results are consistent with the Raman spectroscopy characterization. Eventually, the growth of VACNTs is terminated after deceleration of growth rate due to the deactivation of catalyst particles while fixing the carbon source supply.

Amorphous carbon flakes deposited on the surfaces of catalyst particles, and Al_2_O_3_ substrate will be formed on the bottom of the first layer of VACNTs when the growth of the first layer is terminated, as proved by the results of Electron microprobe. After termination of the growth, the carbon source and the forming gas including hydrogen is continuing aerating. Hydrogen could etch the accumulated carbon on the surface of catalyst nanoparticles randomly [43] and bring carbon source gas into catalyst particles again, turning the deactivated particles into reactivated. Therefore, the catalyst particles function again for the growth of the second VACNT layer, leading to the nucleation and the elongation of the second layer of VACNTs. The extrusion of the new CNT layer is required to release enough mechanical energy to overcome the adhesion energy between amorphous carbon flakes and catalyst/Al_2_O_3_ substrate and then lift the amorphous carbon flakes up, leading to the existence of the carbon flakes at the interface of the top and the bottom CNT layers.

Finite element analysis (FEA) was used to simulate lifting process of amorphous carbon flakes by the extrusion of the second VACNTs growth in the Supplementary Information. Through FEA, it is estimated that 225 nN is the maximum force requested for a CNT to lift up the amorphous carbon film in the same system. It is previously reported that a CNT could bear about 500 nN compression stress during growth, demonstrating our lifting process reasonable in this system [44]. In contrast, the catalyst particle will stay on the surface of the substrate. In the experiments, when the growth time is 15 min, the catalyst is not totally deactivated and no termination of growth happens. However, for growth time of 30 min, both one-layered VACNTs and double-layered VACNTs were observed, indicating the termination and the precipitation of the second-layer CNTs happens at around 30 min growth in the process.

## Conclusions

In summary, we investigated the evolution of the structure of VACNTs with growth time and studied the mechanism for the growth of double-layered VACNTs via single-step CVD process. SEM characterization showed that the termination of the first CNT layer and the precipitation of the second CNT layer happened at around 30 min. Raman spectroscopy analysis showed that the top layer and the bottom layer have similar decreasing quality from the top to the bottom, which indicates that both layers experienced the process of degradation of catalyst particles. Interestingly, as shown by SEM and element analysis, there are carbon flakes at the interface of the two CNT layers. Based on all these observations, a mechanism is proposed, which includes the deactivation of catalyst by accumulation of amorphous carbon, the termination of growth of the first CNT layer, the reactivation of catalyst particles by hydrogen gas, and the precipitation and growth of second CNT layer while lifting the amorphous carbon flakes up. The release of mechanical energy by CNTs provides big enough energy to lift up amorphous carbon coatings on catalyst particles and substrates simulated by finite element analysis. This work helps to get a better understanding of the growth termination of CNTs during a CVD process and may be valuable for the mass production of VACNTs. Besides, the structures of the carbon flakes at the interfaces may benefit the construction of novel three-dimensional carbon structures.

## Electronic supplementary material

Below is the link to the electronic supplementary material.

Supplementary material 1 (PDF 757 kb)
