# Supplementary material for: Investigation on the Formation Mechanism of Double-Layer Vertically Aligned Carbon Nanotube Arrays via Single-Step Chemical Vapour Deposition
Source: Nanomicro Lett. 2016 Oct 7;9(1):12. doi: 10.1007/s40820-016-0113-5 (PMC6223781; doi:10.1007/s40820-016-0113-5)
Supplement: Supplementary file 1 — Supplementary material 1 (PDF 757 kb) [file 40820_2016_113_MOESM1_ESM.pdf]

Supplementary Information for

Investigation on the Formation Mechanism of Double-Layer Vertically Aligned Carbon Nanotube Arrays via Single-Step Chemical Vapor Deposition

Shoumo Zhang[1], Deli Peng[1], Huanhuan Xie[1], Quanshui Zheng[1,3,*], Yingying Zhang[1,2,*]

[1]Center for Nano and Micro Mechanics, School of Aerospace Engineering, Tsinghua University, Beijing 100084, People's Republic of China

[2]Department of Chemistry, Tsinghua University, Beijing 100084, People's Republic of China

[3]State Key Laboratory of Tribology and Applied Mechanics Laboratory, Tsinghua University, Beijing 100084, People's Republic of China

*Corresponding authors. E-mail: zhengqs@tsinghua.edu.cn; yingyingzhang@tsinghua.edu.cn

Tel: +86-13501066263; +86-18210127469

1 Element Finite Analysis

We want to estimate how much load a carbon nanotube can bear when it grows and lifts up the amorphous carbon film covered above. The geometric diagram of the calculation model is showed in Fig. S1.

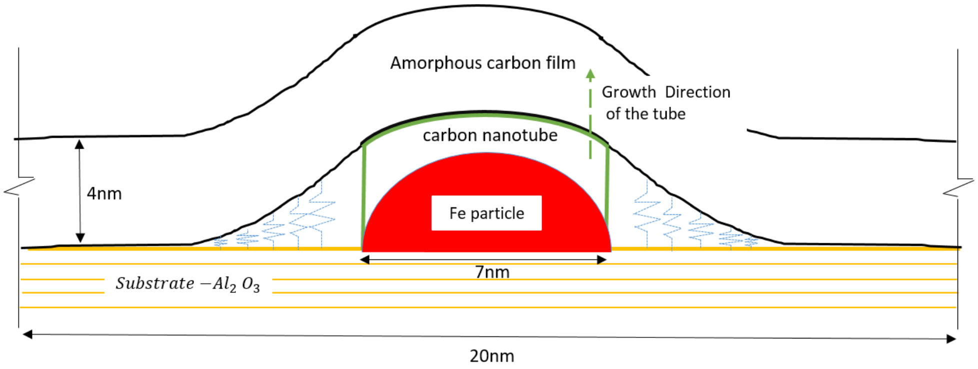

Fig. S1 The plan geometric diagram of the simplified calculation model. The blue spring represents the cohesive relationship between carbon film and substrate

The scale of the model is in the order of 20 nm. However, for the molecular simulation, 20 nm is still a big scale to calculate. Besides, there are few previous data about the potentials of C and Fe and of C and Al_2O_3. Therefore, we choose the finite element simulation to make a preliminary estimate. The Young's modulus and Poisson ratio of amorphous carbon film are E_1 = 759 GPa and v_1 = 0.17 [1]. The substrate can be considered as a rigid body in our estimate. The challenge is how to describe the desorption process of carbon film from the substrate during the growth of the second layer VACNTs in the finite element calculation.

The adsorption energy between graphene layer and Al_2O_3 substrate, -76.4 meV/C-atom, is used to estimate the adsorption energy between the carbon film and Al_2O_3 substrate [2]. The Cs-Fe bond energy, estimating the adsorption energy between the carbon film and Fe catalysis, is 140 meV [3]. Cs means bond-saturated atoms, the Cs-Fe bond energy is weaker than C_{Uns}-Fe bond energy which means bond-unsaturated atoms [2]. The area density of carbon atoms is 38 atom nm^{-2}. The interaction energy mentioned above can be used to calculate the chemisorption energy between amorphous carbon film and the substrate consist of Fe and Al_2O_3, which is much stronger than physical absorption between them. According to our assumption, most of the absorption between them is physical absorption during the carbon deposition process, which is caused by Van der Waals forces. Therefore, we can use the parameters mentioned above to calculate the maximum load a carbon nanotube could bear in our calculation system.

The Van der Waals attraction force increases at the beginning and then decreases when the distance between two atoms exceeds the equilibrium separation, and there is a maximum value of force curve. Thus, the Traction-separation law in Damage mechanics may be used to simulate this process. We build a simple bilinear cohesive mode. As shown in Figs. S2, S3.

Generally, the Van der Waals force is almost reduced to zero when the distance between two atoms is greater than 10 Å. For the convenience of calculation, we assume the distance between point A corresponding to equilibrium separation and point B corresponding to zero Van der Waals force is 10 Å.

Combined with the bilinear cohesive model and adsorption energy, we get the cohesive stress (peak stress), which is 616 Mpa between the carbon film and Al_2O_3 substrate, and 1130 Mpa between the carbon film and Fe particle. The trouble is how to determine the separation δ_0 corresponding to the peak stress, which will affect the stiffness of this model. Fortunately, according to our simulation, when δ_0 is in the range of 0.1 to 0.4 Å, the difference of final results is about 5 %, and it won't affect our conclusion a lot. Therefore, we unify the value of δ_0 to be 1 Å and then we get the Figs. S2, S3.

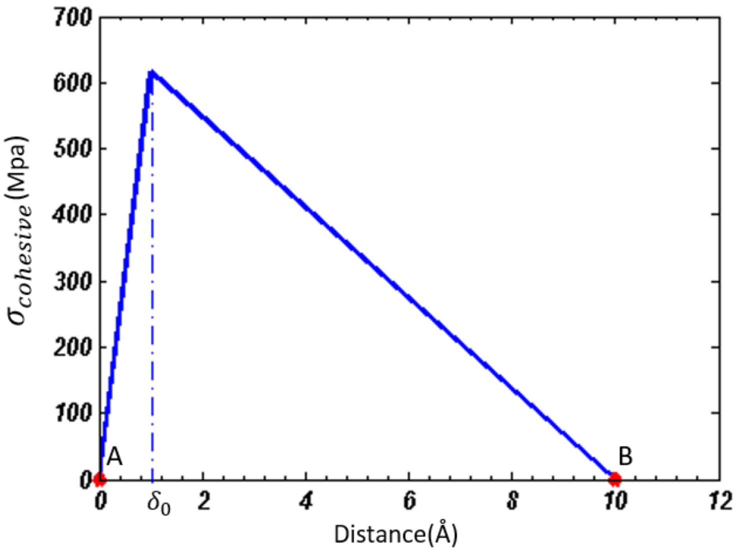

Fig. S2 The cohesive law (bilinear model) of interphase between amorphous carbon film and Al_2O_3 substrate

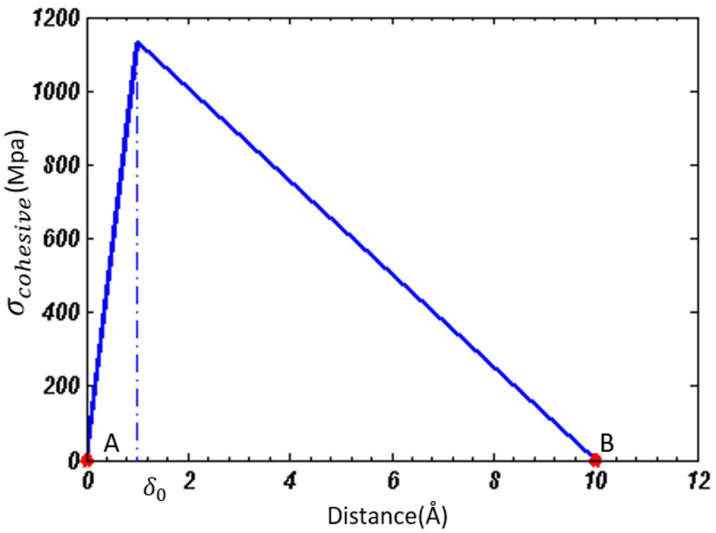

Fig. S3 The cohesive law (bilinear model) of interphase between amorphous carbon film and Fe particle

We then use Abaqus to simulate the process the carbon nanotubes grow and lift up the amorphous carbon film. Cohesive elements are used to realize the cohesive law between the carbon film and substrate. The finite element model is showed in Fig. S4. In Fig. S4, the middle circle with the diameter of 7 nm is used to estimate Fe particle area, while other area of the upper cover is estimating Al_2O_3 substrate. The average diameter of Fe catalyst particles is estimated about 7 nm for multi-wall carbon nanotube [4-7] and the length of one cell unit is 20 nm according to the average CNT densities (2.5×10^{11} CNT cm^{-2}) [8]. The thickness of carbon flake is estimated as 4 nm according to the SEM figures. The displacement load is applied in the middle point of Fe particle area and the force on this point is continuously recorded during the desorption process of carbon film from the substrate until the edges of carbon film are all detached from substrate.

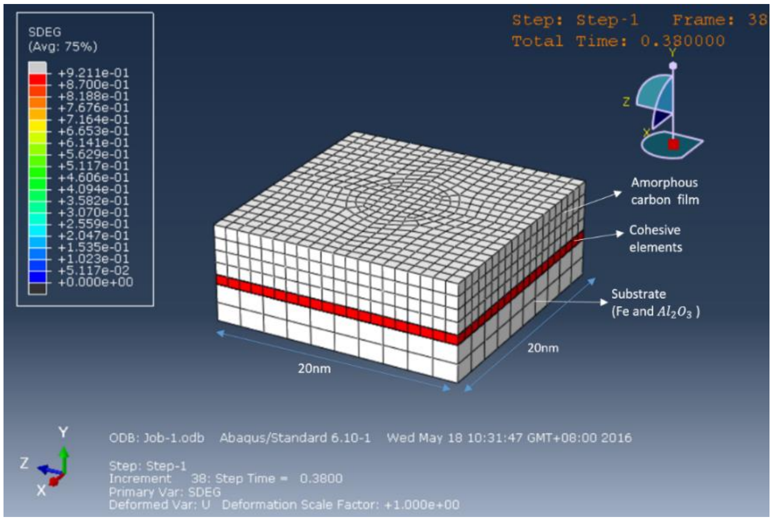

Fig. S4 The finite element model to describe the desorption process of carbon film from the substrate during the growth of one single CNT of the second layer VACNTs. The displacement is loaded at the center area of the bottom surface of the carbon film to simulate the growth of the carbon nanotube

Through this finite element simulation, we get the force curve during adsorption process as is showed in Fig. S5. The maximum load a carbon nanotube could bear is 225 nN.

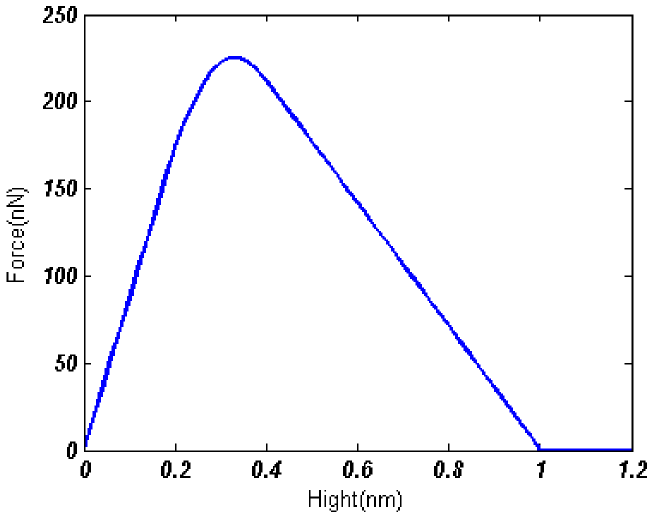

Fig. S5 The force curve of the middle point when one single CNT grows and lifts up the amorphous carbon film from substrate. X label means the growth height of the carbon nanotube and Y label means the force it bears

In this finite element simulation, we simplify the computational process by adopting the bilinear model to simulate the cohesive law between carbon film and substrate and taking the parameters of bonding energy to calculate the adsorption energy caused by Van der Waals forces. The maximum force one CNT could bear during the lifting process is definitely larger than the real force because chemisorption is usually much stronger than physical absorption as mentioned above. However, the purpose of our simulation is to estimate the maximum possible pressure on the carbon nanotubes based on our assumption. The result shows that it can still grow under such pressure, so it won’t affect our final conclusion.

2 A Raman Spectrum

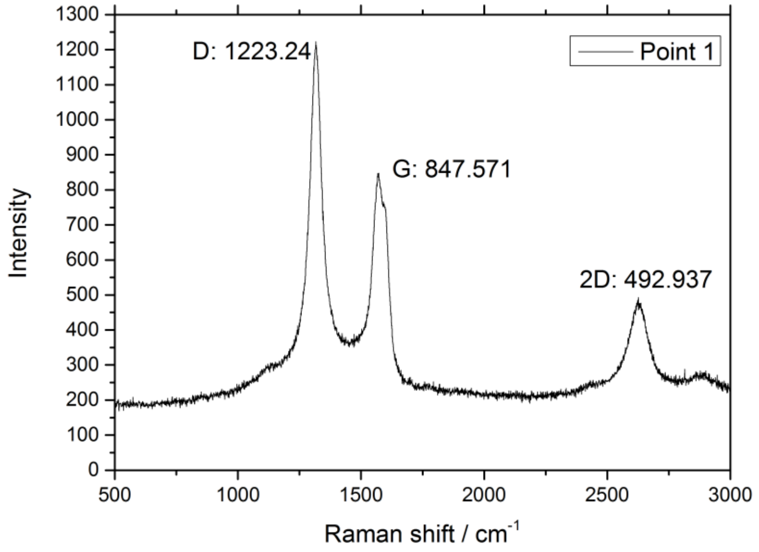

Fig. S6 A typical Raman spectrum taken from the sidewall of VACNTs

References

[1] S. Cho, I. Chasiotis, T.A. Friedmann, J.P. Sullivan, Young's modulus, Poisson's ratio and failure properties of tetrahedral amorphous diamond-like carbon for MEMS devices. J. Micromech. Microeng. **15**(4), 728-735 (2005). doi:10.1088/0960-1317/15/4/009

[2] B. Huang, Q. Xu, S. Wei, Theoretical study of corundum as an ideal gate dielectric material for graphene transistors. Phys. Rev. B **84**(15), 155406 (2011). doi:10.1103/PhysRevB.84.155406

[3] E. Durgun, S. Dag, V.M.K. Bagci, O. Gu¨lseren, T. Yildirim, S. Ciraci, Systematic study of adsorption of single atoms on a carbon nanotube. Phys. Rev. B **67**, 201401(2003). doi:10.1103/PhysRevB.67.201401

[4] P. Pinheiro, M.C. Schouler, P. Gadelle, M. Mermoux, E. Dooryhe'e, Effect of hydrogen on the orientation of carbon layers in deposits from the carbon monoxide disproportionation reaction over Co/Al_2O_3 catalysts. Carbon **38**, 1469-1479 (2000). doi:10.1016/S0008-6223(00)00002-6

[5] K. Hernadi, Catalytic synthesis of multiwall carbon nanotubes from methylacetylene. Chem. Phys. Lett. **363**(1), 169-174 (2002). doi:10.1016/S0009-2614(02)01181-8

[6] K. Hernadi, A. Fonseca, J.B. Nagy, D. Bernaerts, J. Riga, A. Lucas, Catalytic synthesis and purification of carbon nanotubes. Synth. Met. **77**(77), 31-34 (1996). doi:10.1016/0379-6779(96)80051-8

[7] R. Andrews, D. Jacques, D. Quian, E.C. Dickey, Purification and structural annealing of multiwalled carbon nanotubes at graphitization temperatures. Carbon **39**(11), 1681-1687 (2001). doi:10.1016/S0008-6223(00)00301-8

[8] J.K. Holt, H.G. Park, Y. Wang, M. Stadermann, A.B. Artyukhin, C.P. Grigoropoulos, A. Noy, O. Bakajin, Fast mass transport through sub-2-nanometer carbon nanotubes. Science **312**(5776), 1034-1037 (2006). doi:10.1126/science.1126298
